# Internet-of-Things-Enabled Markerless Running Gait Assessment from a Single Smartphone Camera

**DOI:** 10.3390/s23020696

**Published:** 2023-01-07

**Authors:** Fraser Young, Rachel Mason, Rosie Morris, Samuel Stuart, Alan Godfrey

**Affiliations:** 1 Department of Computer and Information Sciences, Northumbria University, Newcastle-upon-Tyne NE1 8ST, UK; 2 Department of Health and Life Sciences, Northumbria University, Newcastle-upon-Tyne NE1 8ST, UK

**Keywords:** gait analysis, computer vision, deep learning, signal analysis, pose estimation, BlazePose, smartphone application

## Abstract

Running gait assessment is essential for the development of technical optimization strategies as well as to inform injury prevention and rehabilitation. Currently, running gait assessment relies on (i) visual assessment, exhibiting subjectivity and limited reliability, or (ii) use of instrumented approaches, which often carry high costs and can be intrusive due to the attachment of equipment to the body. Here, the use of an IoT-enabled markerless computer vision smartphone application based upon Google’s pose estimation model BlazePose was evaluated for running gait assessment for use in low-resource settings. That human pose estimation architecture was used to extract contact time, swing time, step time, knee flexion angle, and foot strike location from a large cohort of runners. The gold-standard Vicon 3D motion capture system was used as a reference. The proposed approach performs robustly, demonstrating good (ICC(2,1) > 0.75) to excellent (ICC(2,1) > 0.90) agreement in all running gait outcomes. Additionally, temporal outcomes exhibit low mean error (0.01–0.014 s) in left foot outcomes. However, there are some discrepancies in right foot outcomes, due to occlusion. This study demonstrates that the proposed low-cost and markerless system provides accurate running gait assessment outcomes. The approach may help routine running gait assessment in low-resource environments.

## 1. Introduction

Running for sport and exercise has continually grown in popularity due to its obvious health benefits and low (cost, skill, resource) barrier to entry [1]. As participation in running has increased, so too has the assessment and analysis of running gait to, e.g., avoid injury or improve efficiency. Running gait assessment generally encapsulates the study of lower-extremity kinematics [2]. Particularly, running gait assessment is paramount in providing injury prevention and rehabilitation mechanisms through quantifying the factors pertaining to the unique patterns of limb movement and co-ordination [3]. For example, runners exhibiting a rear-foot strike have been found to be at almost twice the risk of a repetitive strain injury in comparison to those with a fore or mid-foot strike [4]. As such, identifying and adapting small changes such as strike location to the running stride can minimize injury risk. Additionally, identifying shortcomings and adapting the running stride can lead to performance optimizations and overall better technique and speed. For example, adopting a forefoot strike location can minimize ground contact time, contributing to an increased race speed [5], and improve metabolic economy [6].

Traditionally, running gait assessment has relied upon manual, visual/video-based observation of treadmill running by a trained individual (e.g., bio-mechanist, sports therapist). However, there is some dispute as to the reliability of such approaches between assessors, often being reliant upon the observed plane and/or assessor experience [7]. Consequently, running gait assessment has moved towards instrumented approaches such as wearable technology including inertial measurement units (IMU), force/pressure plate analysis [8,9,10], as well as three-dimensional (3D) motion tracking [11,12] in an effort to provide reliable, reproducible outcomes. Despite research-grade wearable technology’s utility in providing a wide range of gait outcomes, they are currently limited in use due to the cost [13], tethering of peripheral technologies, and reliance upon bespoke environments with expert assistance [9]. For example, wearable sensors or 3D-motion reflective markers often rely upon precise anatomical placement for optimal use [14,15]. Additionally, such technologies are naturally intrusive, often requiring adhesion to the skin or clothes by, e.g., medical tape [16], which may cause discomfort and thus, affect a natural running cycle; limiting the usability of such approaches for habitual use. Consequently, investigation into the use of markerless assessment systems for running gait assessment is warranted.

Markerless systems typically encapsulate human pose estimation methods from 2D or 3D video streams. For example, OpenPose [17] utilizes deep learning-based part affinity fields to identify anatomical locations from a 2D video stream, providing a digital skeleton of an individual performing a range of tasks. Alternatively, Microsoft’s Kinect [18] utilizes depth sensors and infrared cameras to provide a 3D mapping of a human skeleton in near real-time. Although 3D approaches such as Kinect have demonstrated validity in certain spatiotemporal gait outcomes [19], their innate high cost (e.g., specific hardware rather than a generic camera) in comparison with 2D-based deep learning approaches limit their use much like wearable and marker-based tracking technologies. Accordingly, a 2D-based approach is generally preferred (and is the focus of this study).

To date, 2D markerless pose estimation has primarily seen research interest within normal walking/gait assessment. For example, markerless pose estimation has been investigated for use within clinical-based studies [20,21,22], where computer vision-based gait assessment could be used as a primary biomarker to assess disease onset and well-being. In the referenced studies, both spatial [20,21,22] and temporal [21] outcomes are evaluated with generally positive results. In contrast, there are significantly fewer developments for running gait with 2D pose estimation, although the use of OpenPose has demonstrated an ability to examine cadence [23]. Yet, within running gait, there are a significantly higher number of relevant outcomes to inform, e.g., performance optimization such as contact time, swing time, and knee flexion angle [24,25]. Accordingly, this warrants further exploration of the validity of markerless pose estimation within running gait. Furthermore, any contemporary approach to analyzing running gait should ensure flexibility to assess in any environment, beyond bespoke facilities to evoke natural running patterns [26]. Accordingly, reliance upon the OpenPose infrastructure, despite its reliability, requires significant computational cost (e.g., multi-GPU, high-powered computer) and so limits use within habitual or low-resource settings. In contrast, routine technologies (e.g., smartphones) may be more suitable. As such, low-powered 2D approaches must be investigated to enable running gait assessment beyond complex, research-grade environments.

BlazePose is a low-powered markerless pose estimation technique and has recently demonstrated the ability to run on inexpensive hardware (Pixel 2 smartphone) [27]. The approach has been shown to provide a tradeoff relationship with OpenPose, sacrificing some anatomical accuracy for a significant reduction in computational cost in clinical environments [28]. Consequently, the approach could augment assessments within low-resource settings through deployment on low-powered hardware such as a smartphone.

Considering approx. 90% of adults own a smartphone [29], and the technology is generally ubiquitous in everyday life. Accordingly, smartphones are providing a mechanism for remote healthcare, data capture, and transmission within the Internet of Things (IoT) [30] due to their relatively low computational power, connectivity and storage capabilities. Equally, a smartphone can capture data, while offloading complex computations to an external device (e.g., server). Consequently, smartphones could be used to augment complex assessments that were previously reliant on expert in-person visits. Applied to running gait assessment, a smartphone was conceptualized as the data capture mechanism (via video) and then transmitted to a local edge device to enable a more accessible running gait assessment. As such, here, a single-camera approach to running gait assessment utilizing the low-powered BlazePose 2D pose estimation framework is proposed. The study aims to assess the validity of the low-powered approach, running on a custom smartphone application and low-cost server in comparison to a reference-standard 3D motion tracking system. Through validating the low-resource approach, the work aims to contribute to moving the field of running gait assessment out of bespoke facilities (e.g., lab) and into low-resource (e.g., everyday/habitual) environments by providing a simplified mechanism for running gait assessment (smartphone camera) in comparison to existing gold-standard approaches, that could conceivably be used, e.g., in the home, or at the gym.

## 2. Materials and Methods

### 2.1. Participants

Thirty-one healthy, experienced runners (34.5 ± 9.7 years; 1.75 ± 0.30 m; 76.2 ± 4.1 kg; 20 male:11 female) were recruited from running clubs throughout the Northeast of England. Participants were screened for previous running-related injuries (RRI) and their ability to perform unsupervised, short treadmill-based running bouts. No participants reported any gait-affecting injuries or conditions that would adversely affect their ability to participate in the study and all had previous treadmill running experience.

Ethical approval for the study was granted by Northumbria University’s Research Ethics Committee (reference: 21603). Prior to testing, participants were given informed consent and provided verbal and written consent following a short briefing. Upon consenting, participants were provided with a standardized, neutral cushioning running shoe (Saucony Guide Runner) to wear during testing in order to remove bias from gait-affecting cushioning within, e.g., support cushioning running shoes [31].

### 2.2. Video Capture

Participants were video recorded from the side and rear angles during treadmill running sessions utilizing two iPhone 13 smartphones, capturing at 240 frames per second (FPS) to provide slow-motion video streams for the application of 2D pose estimation. The smartphones were placed in separate static mounts approx. six feet from the left side and rear of the treadmill, standardizing the video capture sessions. Before running, participants were filmed performing a short static test, where they stood up straight with their hands to the side to establish a baseline reading. Once calibrated, participants performed up to five 1 min runs on a treadmill between 8 km/h and 14 km/h, selected based upon a pace comparable to their most recent outdoor 5 km pace.

### 2.3. Reference System and Data Labelling

To provide a ground truth to benchmark the system performance against, a 14-camera 3D motion tracking system (Vicon Vertex, UK, www.vicon.com, accessed on 28 November 2022) was used, providing a high-resolution 3D skeletal mapping of participants during treadmill running. Participants were fitted with 16 neo-reflective markers, located at the calcaneal tuberosity (heel), lateral malleoli (ankle), base of the second metatarsal (front-foot/toe), lateral mid-shank, lateral knee joint line, mid-lateral thigh, anterior superior iliac spine, and posterior superior iliac spine. The Vicon 3D motion tracking system was configured to poll at 200 Hertz (Hz) to provide a suitable rate of detail for intricate gait assessment.

To ensure robust outcomes were obtained from the Vicon system, the built-in gait analysis suites were utilized, which have been used to provide a gold-standard set of gait outcomes for comparison with other sensing technologies such as infrared cameras [32] and wearable technology [33]. The following gait outcomes were obtained from the Vicon system.

- Initial contact (IC)—the point at which the foot first contacts the ground.
- Final contact (FC)—the point at which the foot first leaves the ground.
- Contact time (CT)—the total time elapsed between IC and FC (i.e., time foot spent in contact with the ground.
- Swing time (ST)—the time elapsed between an FC event and a proceeding IC (i.e., time foot spent off the ground).
- Step time (StT)—the time elapsed between two IC events.
- Cadence—the number of steps taken per minute of running.
- Knee flexion angle—the angle between lateral mid-shank, lateral knee joint line, and mid-lateral thigh throughout a gait cycle.
- Foot strike location—the angle of the foot during contact with the ground during IC.

### 2.4. Proposed Low-Cost Approach

Typically, OpenPose [17], has been used for 2D computer vision-based running gait assessment but requires a complex GPU configuration due to reliance on convolution pose machines [34] and part affinity fields [35], which may create a barrier of entry to typical users through high associated computational costs. Furthermore, through utilizing part affinity fields, OpenPose performs multi-person pose detection, which creates additional complexities that are unnecessary within running gait assessment (for a single individual). Alternatively, Blazepose provides 33 anatomical key points for single-person videos, which include hips, knees, ankles, heels, and toes. That provides a suitable level of anatomical detail for understanding running gait biomechanics [36], while retaining a substantially lower computational cost by naively relying on identification of the head and its relative position to the body [27]. By utilizing the naïve approach, the BlazePose architecture could in turn augment gait assessment on low-cost hardware such as a smartphone or within an edge computing context, especially when considered against, e.g., OpenPose.

#### 2.4.1. Proposed Infrastructure

**Figure 1:**
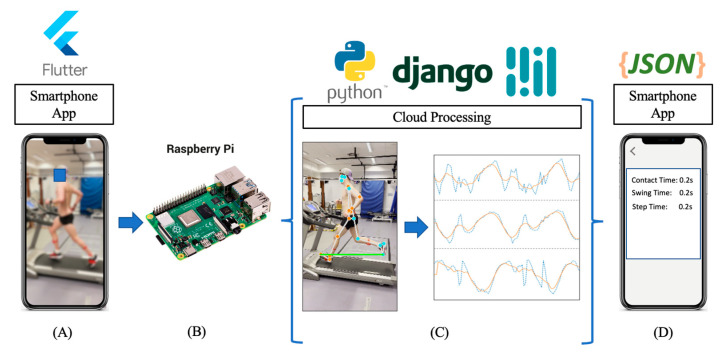
The infrastructure of the smartphone application and its cloud interface. Here, (**A**) the smartphone application captures and uploads side-view videos, transmitting to (**B**) a cloud processing unit running on a Raspberry Pi 4 where a Python Django implementation of Blazepose estimates and (**C**) stores the location of anatomical locations/keypoints, wherein signals are analyzed for gait outcomes. (**D**) Outcomes are then returned to the user via JSON for interpretation in the smartphone application.

To maximize the utility of the proposed 2D approach, a smartphone application was developed, interfacing with a Raspberry Pi server where pose estimation and analysis take place. By utilizing a smartphone in combination with IoT-based edge processing, the system could in turn be adapted for full-scale development/release, lowering the barriers of entry to running gait assessment. An overview of the infrastructure can be found in Figure 1, detailing the flow from a custom smartphone application to the Raspberry Pi-based server, where a cloud processing unit extracts gait outcomes for display on the smartphone application.

#### 2.4.2. Smartphone Application and Cloud Infrastructure

Here, a cross-platform (iOS, Android) smartphone application was developed with the Flutter 2.0 software development kit (SDK) wherein users can capture and upload 1 min (left) side-view videos of treadmill running. Selected videos are compressed using H.264 compression to optimize data transmission to a low-powered cloud infrastructure, wherein analysis takes place.

Despite Blazepose’s ability to perform on-device pose estimation, the approach still requires reasonably modern hardware (e.g., iOS version 12+). As such, a low-powered, low-cost cloud infrastructure was developed utilizing a Raspberry Pi 4 (GBP ≈ £35) and Python’s Django library to maximize the compatibility with a larger number of smartphone devices (Figure 1). The cloud infrastructure first receives videos from the smartphone application and temporarily stores them. Then, the video is run through a BlazePose instance within a Django server, wherein the X and Y locations of relevant anatomical key points of each frame are stored in a .CSV file. The generated .CSV file is then analyzed by the running gait feature extraction layer; wherein extracted outcomes are rendered as a JSON object for transmission to the smartphone application for interpretation, i.e., running assessment. Following a successful analysis, the video is deleted from the cloud storage to maintain user privacy.

### 2.5. Feature Extraction

The feature extraction layer of the proposed system was performed using libraries that are commonplace within Python’s data science and computer vision capabilities (Figure 1). Particularly, OpenCV [37] handles the video stream playback and metadata, where SciPy, Pandas, and NumPy [38,39] were used in combination for data manipulation, signal analysis, statistics, and other mathematical operations detailed throughout the paper. The following section details how running gait features are extracted from the X and Y locations of anatomical keypoints utilizing signal analysis.

#### 2.5.1. Data Preprocessing

Occasionally, keypoints identified by BlazePose can behave erratically and may be subject to noise that is not indicative of the real position of the anatomical location. Consequently, applying a preprocessing layer to the signals is paramount for accurate analysis. Here, a 5-step moving average is applied to each signal stream, optimally selected by manual observation of the signal, ensuring noise is reduced while minimizing loss of signal fidelity.

#### 2.5.2. Gait Mechanics: Identifying Key Features

Initial contact and final contact are key biomarkers within gait as they inform a vast range of temporal and angle gait features. As such, quantifying IC and FC before other features will augment the rest of the gait feature extractions. Within a running stride, IC and FC typically proceed with a maximum extension of the leg [40], wherein the angle between the hip, knee, and ankle is at its maximum value (tending towards 180°), referred to here as the leg extension angle. To calculate the leg extension angle, locations are converted to vectors for vector scalar product analysis such that:

(1) $$\vec{KH}=K-H\;and\;\vec{KA}=K-A$$

where *K* = the location of the knee, *H* = the location of the hip, and *A* = the location of the ankle extracted by the pose estimation layer. $\vec{KH}$ refers to the vector between knee and hip, whereas $\vec{KA}$ refers to the vector between knee and ankle, with *K* being the intersecting point of two vectors. Proceeding vector conversion, the scalar product of the two intersecting vectors can be represented as:

(2) $$\vec{KH}\cdot \vec{KA}=\left|\vec{KA}\right|\left|\vec{KH}\right|cos\left(\theta \right)$$

Here, e.g., $\left|\vec{KH}\right|$ refers to the magnitude of the vector between the knee and hip such that:

(3) $$\left|\vec{KH}\right|=\sqrt{{\left(K_{x}-K_{y}\right)}^{2}+{\left(H_{x}-H_{y}\right)}^{2}}$$

and *θ* is the angle between the intersecting vectors. Consequently, the following expression can be solved for *θ* such that:

(4) $$\theta =cos^{-1}\left(\frac{\vec{KH}\cdot \vec{KA}}{\left|\vec{KA}\right|\left|\vec{KH}\right|}\right)$$

**Figure 2:**
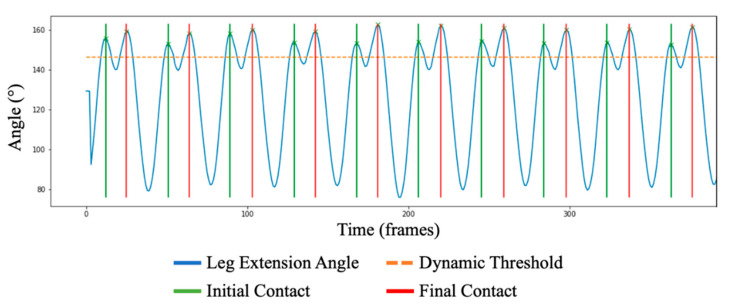
The identification of IC and FC, quantified by observing peaks within the leg extension angle data stream. Here, the dynamic threshold has been set at 90% of the maximum observed angle, wherein peaks are detected above the threshold. As seen, there is a significant angle reduction before an IC, with a smaller reduction before FC; providing distinguishable features between the two.

With the angle between the hip, knee, and ankle calculated, IC and FC are calculated in the following manner:

1. A dynamic threshold is set at the 90th percentile of the maximum leg extension angle within the signal.
2. A zero-crossing gradient maxima peak detection algorithm such as those found within other gait assessment applications, e.g., [41] detects two peaks above the dynamic threshold, IC and FC.
3. IC and FC are then distinguished apart by observing the minima of the signal prior to the identified peak. Before an IC, the signal will dip significantly lower than the signal prior to FC due to minimum leg extension (i.e., lowest angle between hip, knee, and ankle; highest flexion) during the swing phase of gait, opposed to a maximum extension during contact [40] (Figure 2).

#### 2.5.3. Temporal Outcomes and Cadence

**Figure 3:**
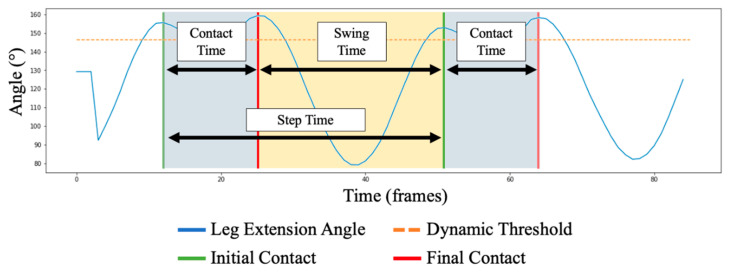
The following figure shows a magnified view of Figure 2. Here, an illustration of the calculation of temporal running gait outcomes is presented. CT is shown as the time between an IC and proceeding FC, ST is shown as the time between FC and a proceeding IC; whereas StT is the time between two respective IC events.

Upon the identification of IC and FC; CT, ST and StT can be calculated such that:

(5) $$\mathrm{CT}=(FC_{x}-IC_{x})/FPS$$

(6) $$\mathrm{ST}=(IC_{x+1}-FC_{x})/FPS$$

(7) $$\mathrm{StT}=(IC_{x+1}-IC_{x})/FPS$$

where *x* refers to the currently observed stride (e.g., *x* + 1 refers to the next stride). Values are then normalized from frames (*FPS*) into seconds for user-interpretable outcomes (Figure 3). Proceeding this, cadence is then quantified by first calculating the average number of steps per second × 60 s.

#### 2.5.4. Knee Flexion Angle

Utilizing the leg extension angle calculation detailed as part of Section 2.5.2, the knee flexion angle can be calculated as a natural byproduct. Here, the knee flexion angle is recorded during an identified IC event, during an identified FC event, as well as the minimum value within a single step (i.e., within two ICs). Knee flexion angle is also stored continually throughout strides for observational comparison between estimated and ground truth angles.

#### 2.5.5. Foot Strike Angle and Location

As previously described, foot strike location refers to the location of the foot that makes contact with the ground during IC, often referred to by rearfoot, midfoot, and forefoot striking patterns [42]. As such, calculating the angle of the foot is necessary. The angle of the foot is calculated between the toe, heel, and a point placed directly vertical from the heel (vH), where the angle is calculated using the vector scalar product as above (Section 2.5.2), with hip, knee, and ankle replaced with toe, heel, and vH, respectively.

During the calibration period in data capture, wherein participants stood still for a short time before running, a baseline reading of the foot angle is taken to account for any offset experienced during video capture (e.g., horizontal recording may not be perfectly aligned with treadmill, therefore it cannot be assumed that the resting foot lies at 90°). After calibration, the foot strike angle is then recorded during an identified IC event. An average angle is taken throughout all identified strikes and compared against the baseline reading. Foot strike location is then described as: >5° from baseline = forefoot; <−5° from baseline = rearfoot; and between −5° and 5° from baseline = midfoot strike patterns.

### 2.6. Statistical Analysis

The statistical analysis examining the performance of the proposed approach was performed in SPSS v27. Shapiro–Wilk tests indicated a parametric distribution for CT, ST, knee flexion angle, and foot strike angle; and non-parametric distribution for StT. As such, a log_10_ transformation was applied to non-parametric data to allow for Pearson’s correlation scores to be used throughout the outcomes. Additionally, to examine the reliability between 3D motion tracking and pose estimation approaches, intra-class correlation (ICC_(2,1)_) was deployed, examining the absolute agreement between the measures. ICC_(2,1)_ performance was defined in line with the accepted performance guideline such that the result can be described as poor (<0.500), moderate (0.500–0.750), good (0.750–0.900), and excellent (>0.900) [43]. Additionally, mean error, Bland–Altman, and box plot analysis were used to provide a visual assessment of the performance of the system in comparison to ground truth 3D motion tracking data.

## 3. Results

No data dropout or loss occurred throughout the data capture process, resulting in the full range (*n* = 31) participants being utilized in the results. In total, 148 running bouts were captured (between 4 and 5 running bouts per participant), observing 9327 unique strides during the proposed study. (Here, accuracies for all runs are presented, see Supplementary Materials for results breakdown across all running speeds.)

### 3.1. Temporal Outcomes

**Figure 4:**
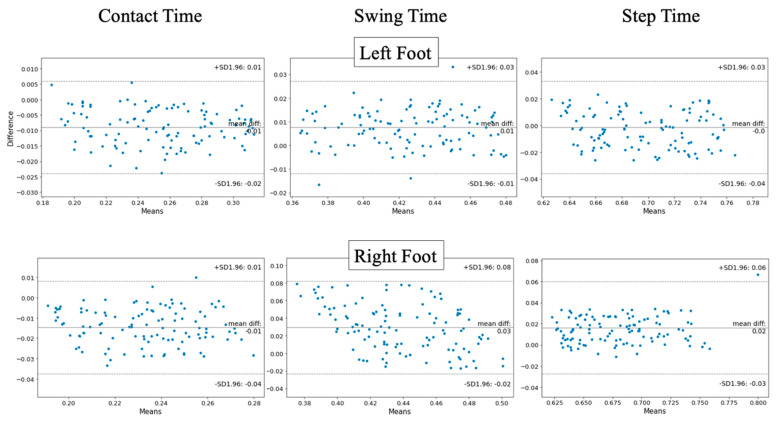
Bland–Altman plots illustrating the mean error between BlazePose estimation and reference standard 3D motion tracking data streams for contact time, swing time, and step time between left and right feet.

**Figure 5:**
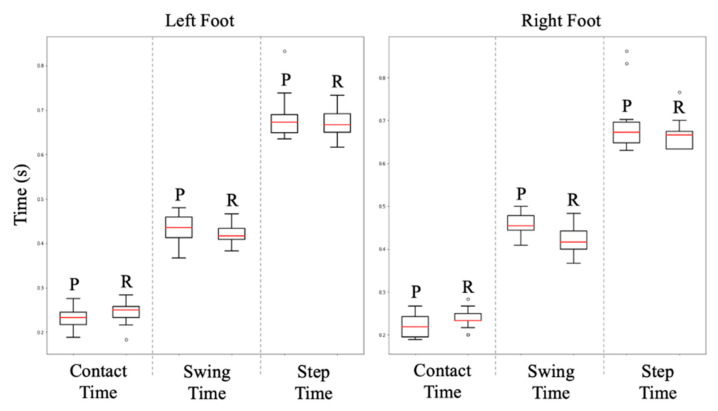
Box plots illustrating the temporal difference between BlazePose predicted (**P**) and reference (**R**) data streams for contact time, swing time, and step time at all speeds, observing the differences between left and right foot outcomes.

For all running speeds, intraclass correlation demonstrates good (ICC_(2,1)_ ≥ 0.751) agreement across CT, ST, and SwT features. This indicates results obtained from 2D pose estimation are strongly indicative of measures obtained from the gold standard 3D motion tracking approach. Additionally, the features observe relatively low mean error (left foot 0.011–0.014 s; right foot 0.014–0.033 s) from 3D motion tracking-produced outcomes (Table 1). Figure 4 and Figure 5 containing Bland–Altman and box plots (respectively) illustrate the performance of each measure. Accuracy of pose estimation outcomes is almost unanimously higher within the left foot in opposition to the right, with right foot swing time and step time measures tending to overestimate outcomes.

### 3.2. Cadence

The proposed approach to cadence identification demonstrates excellent intraclass correlation scores (ICC_(2,1)_ = 0.981) across the range of tested running bouts. With a mean error rate of 1.2 steps, pose estimation can accurately estimate cadence in comparison to reference tracking data.

### 3.3. Knee Flexion Angle

**Figure 6:**
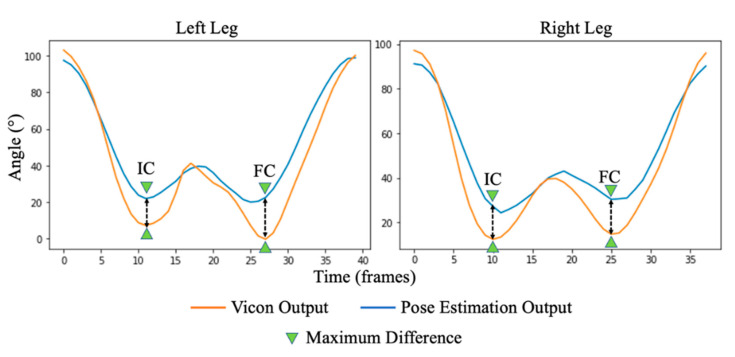
An illustration of the knee flexion angle of an average stride within the dataset between left and right legs. Here, it can be seen that pose estimation (orange) can closely match 3D motion tracking (blue) above ~40°; however, a maximum difference is exacerbated below ~40°, indicating pose estimation may overestimate knee flexion angles during maximum extension events (IC/FC).

Here, the performance score is derived from the average measure experienced across an accumulation of all strides. ICC_(2,1)_ scores indicate excellent (left leg ICC_(2,1)_ = 0.961; right leg ICC_(2,1)_ = 0.979) correlation between data streams obtained between pose estimation and 3D motion tracking outcomes, with no significant deviation between left and right legs. However, when observing the average obtained waveforms (Figure 6) there is a widening distance between the measures surrounding IC and FC events, wherein the pose estimation approach overestimates the knee angle at the point of maximum extension.

### 3.4. Foot Angle and Foot Strike Location

Assessment of foot angle and strike location is separated to assess the performance of the pose estimation approach throughout both (i) the entire stride and (ii) at impact (i.e., providing foot strike location), providing a rearfoot, midfoot, or forefoot strike classification.

#### 3.4.1. Foot Angle

**Figure 7:**
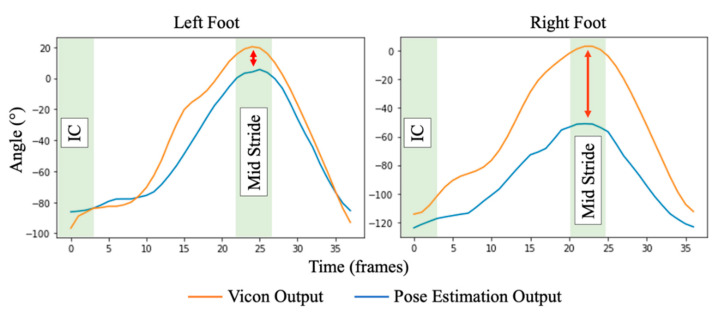
An illustration of the foot angle of an average stride within the dataset between left and right feet. Here, there is a significant deviation between left and right feet. In the left foot, signals are closely associated with surrounding initial contact events and begin to slightly deviate during the mid-stride. Alternatively, the right foot demonstrates a further deviance between pose estimation and 3D motion tracking signals, exacerbated during the mid-stride.

Throughout the stride, outcomes demonstrate excellent (ICC_(2,1)_ = 0.981) and good (ICC_(2,1)_ = 0.844) correlation between pose estimation and 3D motion capture outcomes for left and right feet, respectively. Observing waveforms obtained from pose estimation and 3D motion capture systems and min/max values (Figure 7 and Table 1), the pose estimation approach consistently underestimates foot angle in both left and right feet. In both, and particularly within the right foot, a significant underestimation of foot angle is experienced as the angle tends towards positive. However, the foot angle is significantly closer surrounding the location of a typical IC event, where foot angle primarily affects gait.

#### 3.4.2. Foot Strike Location

Classifications were measured based on each participant’s baseline resting foot angle, measured within video stream calibration. Accuracy of the foot strike location (i.e., forefoot, rearfoot and midfoot) demonstrated 95.2% and 89.8% in left and right feet, respectively. This indicates that despite deviance in foot angle throughout the stride, foot strike location can be accurately assessed utilizing BlazePose estimation.

## 4. Discussion

Understanding running gait is crucial in performance optimization and providing mechanisms for injury prevention and rehabilitation [4]. Providing low-cost mechanisms to measure running gait beyond bespoke settings is important to enabling accessibility and assessments for a wider range of individuals. This study has demonstrated the viability of a lightweight pose estimation, smartphone camera-based approach to provide running gait assessment.

### 4.1. Pose Estimation Performance

The single-camera BlazePose approach demonstrates high performance in both angle and temporal running gait outcomes in comparison to a Vicon 3D motion tracking reference system. Particularly, ICC_(2,1)_ scores throughout the range of extracted outcomes indicate good to excellent agreement with reference streams; whereas temporal outcomes maintain a low mean error.

#### 4.1.1. Temporal Outcomes

In comparison to similar studies within the field utilizing computer vision techniques, the proposed approach performs comparably within temporal gait outcomes, despite operating from a smartphone camera. For example, Garcia-Pinillos et al. [44] utilized the Microsoft Kinect system to extract contact and swing time, compared against both the OptoGait and high-speed video reference streams. In the study, the Kinect-based approach can extract temporal running gait features with an ICC of 0.712 and 0.894 in comparison to OptoGait; and 0.667 and 0.838 in comparison to high-speed video streams for contact and swing time, respectively. In comparison to the proposed approach exhibiting ICC scores of 0.862/0.861 (left/right) and 0.837/0.821 (left/right) for contact and swing time, 2D pose estimation can extract temporal gait outcomes comparably to approaches using dedicated hardware such as the Kinect.

There is a small effect on the performance of the temporal feature extraction layer between slow (8 km/h) and fast (14 km/h+) running speeds, with a slightly observably lower agreement and higher mean error experienced in higher speeds in comparison to those in lower speeds (Supplementary Materials). The higher mean error in faster speeds could be attributed to the video frame rate provided by the smartphone camera (60 FPS) not providing a suitable resolution to distinguish the naturally shorter temporal outcomes associated with faster running [45]. In fact, it has been shown that producing reliable gait outcomes at higher speeds (14.4 km/h+) will require up to 100FPS video capture streams [46]. Consequently, further investigation into the effects of frame rate (e.g., 120/240 FPS) on the pose estimation approach may be necessary to move into, e.g., elite running speeds, where the likes of contact time could be significantly shorter than those observed within this study.

Although the approach generally exhibits a low mean error from reference streams, there is an observably lower agreement in comparison to studies utilizing wearable technology such as IMU sensors. For example, a foot-mounted IMU has demonstrated an ability to extract contact and swing time with excellent (>0.9) agreement with reference streams [47]. The reduction in performance could be dependent on lighting. Particularly, fluctuations of light within a scene can greatly affect the pixel values, effectively creating noise within an image [48], which may hinder the performance of pose estimation frameworks. Additionally, wearable sensors can be configured to capture at a faster rate (e.g., 100 Hz–200 Hz [49]) which would provide considerably higher resolution for temporal gait outcomes, which can be relevant within 1/100 thousands of a second.

#### 4.1.2. Knee Flexion and Foot Strike Angle

**Figure 8:**
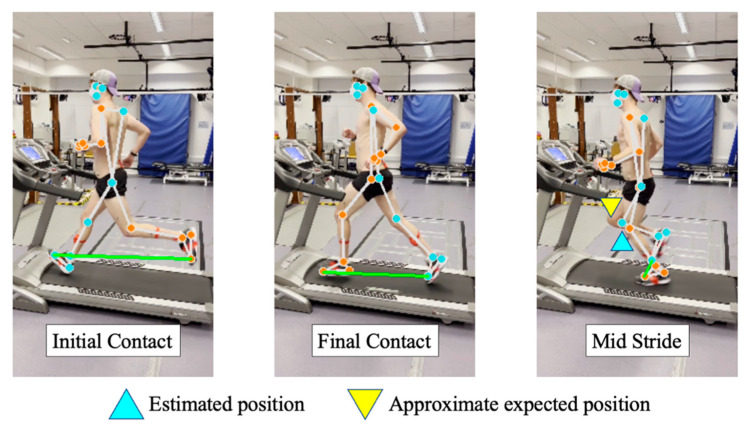
An example of pose estimation output at three different stages of the gait cycle, (i) initial contact, (ii) final contact, and (iii) mid-stride. As shown, during non-occluded segments of the gait cycle (IC/FC), estimated anatomical locations closely resemble their expected positions. However, observing mid-stride (during occlusion between left/right legs) one can begin to see the estimated anatomical locations deviate from expected positions on the right leg (e.g., knee), providing insight behind lower accuracy experienced by right leg gait outcomes.

Observing waveforms and agreement measures of knee flexion and foot angle, the pose estimation approach closely follows the 3D motion tracking reference stream. Within foot angle, the pose estimation approach tends to underestimate the angle, whereas within knee flexion angle the inverse is true, with the pose estimation approach overestimating the angle during maximum extension periods. This is likely due to the camera placement relative to the treadmill. For example, in Figure 8, it is evident that the treadmill is not always straight within the sagittal plane, i.e., at 90° to the treadmill. As such, the angles obtained will naturally deviate in comparison to 3D motion tracking, which uses multiple infrared cameras to provide a more accurate angle estimation.

Prevalently within foot angle measures, there is a noticeable reduction in performance experienced between left and right measures (Table 1 and Figure 7). Upon investigation, there were bouts of noise experienced from the pose estimation output where the left leg (facing towards the camera) occludes the right leg (always facing away from the camera), an issue commonly found within pose estimation accuracy and validity measures [50]. Particularly, this provides an explanation for the significant deviance in the right foot angle surrounding the mid-stride, with the left and right legs intersecting throughout the mid-stride (Figure 8). Some studies have begun to address occlusion within the wider pose estimation domain with varying rates of accuracy [51,52,53]. However, the approaches generally rely on expensive computations (requiring complex hardware), which may limit the routine deployment of the system. As such, within the constraints of the study, higher accuracy may be achieved pragmatically by capturing videos from both sides (i.e., right leg at front) and running separate inferences, rather than the inclusion of a potentially expensive occlusion dampening approach.

Despite deviance between left and right feet, the pose estimation approach performs comparably with IMU-based approaches for foot strike location. For example, Young et al. [54] validated an IoT-enabled foot-mounted IMU polling at 60 Hz for the extraction of foot strike location during running gait at 94.3% accuracy. These results are comparable to those obtained within the proposed study, with 95.2% and 89.8% accuracy obtained through analyzing pose estimation data streams. The foot strike location accuracy is largely unaffected by deviation in the angle between estimated and reference streams due to simply observing the angular change surrounding impact. For example, rather than measuring the angle of the foot during impact (which may be inaccurate compared to reference streams), it is more pragmatic to assess the angular change between heel and toe, e.g., if the toe is moving down in comparison to the heel during impact, it is assumed a heel strike has occurred.

## 5. Limitations and Future Work

Currently, there is some discrepancy between left- and right-footed outcomes due to stationary camera location (left side only). This resulted in occlusion experienced when the left leg (front) obstructs the right leg (back). Future work will assess the approach utilizing combined videos from both sides (left and right foot forward) or a moving camera to examine whether a multi or panning video-based approach would improve the occluded discrepancies.

The data capture sessions took place in a well-lit, tailored laboratory which isn’t necessarily indicative of habitual environments that may have, e.g., dimmer lighting or lower-quality smartphone camera equipment. As such, evaluating the systems performance in a range of low-resource environments (including outside) in comparison to, e.g., IMU-based outcomes, will help to validate the approach as a habitual running gait assessment tool.

Finally, spatial outcomes could be incorporated within the proposed system to provide greater utility within running gait assessment. For example, stride length has been shown to affect oxygen uptake [55] and shock attenuation [56] during running, and as such, can be optimized for running performance and injury mechanisms. Measuring stride length will require testing the system on an instrumented treadmill and during overground runs to assess the step/stride and total distance. Consequently, future work will aim to expand the functionality of the approach through use in overground settings, while expanding the suite of gait outcomes.

## 6. Conclusions

The proposed work demonstrates the efficacy of a low-powered pose estimation framework (BlazePose) to provide running gait outcomes within an IoT context. By operating from a smartphone interfacing with an edge device (Raspberry Pi-based server), the approach could contribute towards moving running gait assessment out of bespoke environments and into everyday/habitual settings. The approach exhibits good to excellent (ICC_(2,1)_ 0.751–0.981) agreement for temporal outcomes, cadence, and knee angles compared with reference standard 3D motion tracking. Foot strike angle accuracy was %. Furthermore, the approach demonstrates low mean error with <0.014 s and <0.033 s for left and right streams, respectively. The approach may help routine running gait assessment in a low-resource environment.

## Figures and Tables

**Table 1:** Merged intraclass correlations (ICC_(2,1)_), Pearson’s correlation, mean and mean error scores for the range of outcomes for all running speeds obtained from the proposed pose estimation (predicted) approach in comparison with 3D motion tracking reference data. In addition, min/max predicted and reference knee flexion angle and foot strike location are presented.

Left Foot						
Outcome	Mean Predicted	Mean Reference	Mean Error	ICC_(2,1)_	*r*	
Contact Time (s)	0.232	0.243	0.011	0.862	0.858	
Swing Time (s)	0.434	0.420	0.014	0.837	0.883	
Step Time (s)	0.682	0.671	0.010	0.811	0.845	
	**Min** **Predicted**	**Max** **Predicted**	**Min** **Reference**	**Max** **Reference**	**ICC_(2,1)_**	***r***
Knee Flexion (°)	19.9	98.9	1.4	103.1	0.961	0.975
Foot Strike Loc. (°)	−86.2	5.6	−96.7	20.3	0.981	0.980
**Right Foot**						
**Outcome**	**Mean** **Predicted**	**Mean** **Reference**	**Mean** **Error**	**ICC_(2,1)_**	***r***	
Contact Time (s)	0.220	0.239	0.014	0.861	0.854	
Swing Time (s)	0.457	0.423	0.033	0.821	0.781	
Step Time (s)	0.689	0.665	0.024	0.751	0.769	
	**Min** **Predicted**	**Max** **Predicted**	**Min** **Reference**	**Max** **Reference**	**ICC_(2,1)_**	***r***
Knee Flexion (°)	30.4	91.1	12.5	97.1	0.979	0.982
Foot Strike Loc. (°)	−124.0	−51.0	−114.4	3.3	0.844	0.911

## Data Availability

The dataset used within the study is not available for use by the public due to identifiable features within video and data streams.

## Supplementary Materials

The following supporting information can be downloaded at: https://www.mdpi.com/article/10.3390/s23020696/s1, Supplementary material contains a breakdown of results for proposed system across all running speeds.
